# Neuroprotective Effects of Cannabispirenone A against NMDA-Induced Excitotoxicity in Differentiated N2a Cells

**DOI:** 10.1155/2024/3530499

**Published:** 2024-02-13

**Authors:** Sonia Thapa, Yedukondalu Nalli, Ajeet Singh, Shashank Kr. Singh, Asif Ali

**Affiliations:** ^1^Cancer Pharmacology Division, CSIR-Indian Institute of Integrative Medicine, Canal Road, Jammu 180001, India; ^2^Academy of Scientific and Innovative Research (AcSIR), Ghaziabad 201002, India; ^3^Natural Products Chemistry Division, CSIR–Indian Institute of Integrative Medicine, Canal Road, Jammu Tawi 180001, India; ^4^Medicinal and Process Chemistry Division, CSIR-Central Drug Research Institute, Lucknow 226031, India

## Abstract

The endocannabinoid system is found throughout the central nervous system, and its cannabinoids receptor 1 is critical in preventing neurotoxicity caused by N-methyl-D-aspartate receptor activation (NMDARs). The activity of NMDARs places demands on endogenous cannabinoids to regulate their calcium currents. Endocannabinoids keep NMDAR activity within safe limits, protecting neural cells from excitotoxicity. Cannabinoids are remembered to deliver this outcome by repressing presynaptic glutamate discharge or obstructing postsynaptic NMDAR-managed flagging pathways. The endocannabinoid system must exert a negative influence proportional to the strength of NMDAR signaling for such control to be effective. The goal of this paper is to draw the attention towards the neuroprotective mechanism of constituents of *Cannabis sativa* against NMDA-induced excitotoxic result. Phytochemical investigation of the cannabis flowers led to the isolation of nine secondary metabolites. A spiro-compound, Cannabispirenone A, which on treatment of the cells prior to NMDA exposure significantly increases cell survival while decreasing ROS production, lipid peroxidation, and intracellular calcium. Our findings showed that this compound showed neuroprotection against NMDA-induced excitotoxic insult, has antioxidative properties, and increased cannabinoid receptor 1 expression, which may be involved in the signaling pathway for this neuroprotection.

## 1. Introduction

Excitotoxicity is caused by glutamate receptor activation, which is one of the main mechanisms leading to cell death. It affects all subcellular compartments, with changes in the cytosol, mitochondria, endoplasmic reticulum (ER), and nucleus. The primary causes of excitotoxicity are increased calcium influx buildup within the cell [1]. During oxidative stress, such as tramautic brain injury or excitotoxic insult, NMDAR channel opens that leads to impaired transport of glutamate as well as reduce glutamate clearance. The reduction in glutamate clearance repeatedly stimulate the NMDAR activation. This process damages neurons, results in intracellular calcium rise across the plasma membrane [2, 3]. Rise in intracellular calcium damages dendrites and kills neurons in part by activating cysteine proteases known as calpains, which degrade a wide range of substrates such as cytoskeletal proteins, membrane receptors, and metabolic enzymes [4, 5]. Reactive oxygen species (ROS) such as superoxide anion radical, hydrogen peroxide, and hydroxyl radical are also produced during NMDA-induced oxidative stress by activating cyclooxygenases and lipoxygenases, disrupting mitochondrial metabolism, and inducing membrane lipid peroxidation [6, 7]. NMDA receptors, receptor-gated ion channels, are important excitotoxic receptors found in the cerebral cortex and hippocampus. NMDA receptors are critical for the development of central nervous system activities as it mediate neural transmission that involves learning, memory, and cognition and have high calcium permeability. Unfortunately, dysfunction of these receptors caused by excitotoxicity by their excessive activity coexist with a number of neurological diseases. Thus, one potential therapeutic approach can be provided by the modulation of NMDAR by specific G-protein coupled receptors (GPCRs). NMDAR and GPCRs both act to regulate each other's signaling and also stimulate intricate cellular signaling networks [8, 9]. The endocannabinoid system (ECS) in this situation contributes significantly to the reduction of NMDAR activity through the activation of cannabinoid receptor 1 (CB1R). The ECS is highly spread as well as highly expressed in the brain. It consists of two GPCRs, namely cannabinoid receptor 1 (CB1R) and cannabinoid receptor 2 (CB2R). CB1R is one of the most prevalent GPCRs in the mouse and human brains, but CB2R is mostly expressed in the microglial cells. One of the best pharmacological functions of CB1R is the inhibition of synaptic activity by reducing the neurotransmitter release. The endocannabinoid system regulates the activity of NMDARs, preventing its overactivation and protecting neuronal cells from excitotoxicity. Therefore, dysregulated NMDAR function could be restored by pharmacologically manipulating this endogenous system. NMDAR activity provokes endocannabinoid release and cannabinoid receptor stimulation, reducing its activity and preventing excitotoxicity. Another possibility is that cannabinoids prevent endogenous calcium influx through mechanisms associated with direct NMDAR channel inhibition [10, 11].

In our earlier studies on *Cannabis sativa*, we have isolated and characterized a new prenylspirodinone with antimicrobial potential, reported the role of cannabinoids as modulators of Wnt/*β*-catenin signaling pathway, and synthesized the cannabidiol derivatives with cytotoxic potentials [12–14]. Continuing our search for bioactive secondary metabolites of the *Cannabis* plant, here, in this study, we isolated 10 secondary metabolites from flowers of *Cannabis sativa* and reported their neuroprotective effect using the differentiated Neuro 2a cell line as an experimental model. In summary, this study reveals the mechanism of Cannabispirenone A (9) that explains how it provides protection against the excitotoxic insult.

## 2. Materials and Methods

### 2.1. Reagents

HPLC grade methanol and acetonitrile were purchased from Merck. DMEM, retinoic acid (RA), penicillin salt, streptomycin salt, sodium pyruvate, PBS, NMDA, BSA, Triton X-100, sodium bicarbonate, protein ladder, N-methyl-D-aspartate receptor (NMDA), H2DCFDA dye, anti-CB1antibody, and *β*-actin were purchased from Sigma (Saint Louis, MO, USA). Fluo-8 AM was purchased from Abcam. Rh-123 dye was purchased from Invitrogen (USA). Bax, Bcl-2, antirabbit, and antimouse antibody HRP linked were purchased from Cell Signaling Technology (USA). PVDF membrane and Immobilon Western Chemiluminescent HRP substrate were purchased from Merck Millipore (Darmstadt, Germany).

### 2.2. General Experimental Procedures

NMR measurements (^1^H, ^13^C, and DEPT-135) were recorded on a 400 and 500 MHz spectrometer fitted with a pulse-field gradient probe, and tetramethylsilane (TMS) or the residual resonance of the deuterated solvent was used as internal reference. Chemical shifts are expressed in ppm, and coupling constants *J* are expressed in Hz. High-resolution mass spectral data were obtained from a Q-TOF mass spectrometer-coupled LC system. Analytical and semipreparative HPLC purifications were carried out on an Agilent HPLC, with an Eclipse XDB-C-18 (5 *µ*m, 250 × 9.4 mm) column, with quaternary pump, photodiode array detector, and auto injector function (Agilent 1260 series).

### 2.3. Plant Material, Extraction, Isolation, and Characterization of Compounds

Authenticated plants (*Cannabis Sativa*) were collected from CSIR—Indian Institute of Integrative Medicine-Campus, Jammu & Kashmir (J&K). The flowers of the plants were air dried and then grounded to powder. These grounded flowers of *Cannabis sativa* (0.6 kg) was sequentially extracted with hexane and chloroform at room temperature to yield 51.7 g and 19.2 g of crude extracts, respectively. Both extracts were combined (70.9 g), subjected to fractionation in an open column using the neutral alumina (Al_2_O_3_) column with gradient elution from hexane to ethyl acetate (0%–100%), and finally eluted by methanol resulting 420 fractions (Fr.1–Fr.200). Fractions from 291 to 309 (0.929 g; eluted from 20% to 30% ethyl acetate) were pooled based on the similar TLC profile and passed through Sephadex LH20 with methanol resulting in an enriched fraction (0.139 g), which was further purified by HPLC chromatography (A: Water, B: Acetonitrile; Agilent Eclipse XDB-C18, 5 *µ*M, 9.5 × 250 mm; flow rate 2 mL/min). Here, 20% B isocratic elution afforded six major peaks: (1) (3.1 mg, *t*_R_ 6.4 min), (2) (7.7 mg, *t*_R_ 8.98 min), (3) (2.3 mg, *t*_R_ 10.52 min), (4) (29.6 mg, *t*_R_ 12.60 min), (5) (12.0 mg, *t*_R_ 14.70 min), and (6) (7.9 mg, *t*_R_ 16.4 min) (Figures S4 and S5). Fractions 320–338 (1.609 g; eluted at 40% ethyl acetate) showing thee major spots on TLC along with chlorophyll were passed through Sephadex LH20 using methanol as an eluting solvent providing two subfractions Fr. A (0.175 g) and Fr. B (0.418 g). Pure compounds **7** (174.5 mg), **8** (76.0 mg), and **9** (58.3 mg) have been isolated from Fr. B fractionated by Silica gel column chromatography eluting with hexane and ethyl acetate (from 0% to 100% EA). The isolated compounds are then characterized based on the HR-ESIMS data, NMR spectroscopic analyses (Figures S1–S3, supporting information), and the comparison of the reported NMR data.

### 2.4. Screening of the Compounds

**Figure 1:**
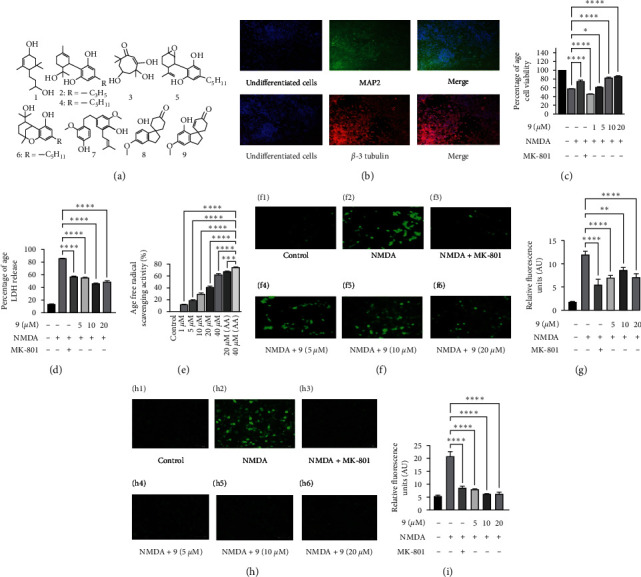
(a) Structures of the compounds isolated from the flowers of the *Cannabis sativa*. (b) Treatment of Neuro 2a cells with retinoic acid (RA) to induce differentiation and expression of MAP2 and *β*-3 tubulin, by immunofluorescence studies. (c) Effect of the molecule (9) on cell viability against NMDA-induced toxicity and (d) LDH release reversal in NMDA-induced toxicity. (e) Free radical scavenging activity of the molecule (9) in a cell-free assay. (f) Effect of the molecule on intracellular calcium overload in NMDA-induced toxicity. (f1) Control, (f2) only NMDA, (f3) MK-801, (f4) NMDA + molecule (5 *µ*M), (f5) NMDA + molecule (10 *µ*M), and (f6) NMDA + molecule (20 *µ*M). (g) Bar graph showing densitometry analysis of the fluorescence obtained. (h) Effect of the molecule on generation of reactive oxygen species (ROS) in NMDA-induced toxicity. ROS were captured via live cell imaging. (h1) Untreated, (h2) only NMDA, (h3) NMDA + MK-801, (h4) NMDA + molecule (5 *µ*M), (h5) NMDA + molecule (10 *µ*M), and (h6) NMDA + molecule 20 *µ*M. (i) Bar graph showing densitometry analysis of the fluorescence obtained. Data represent an average of three independent experiments. Statistical significance was determined using one-way ANOVA analysis followed by Dunnett's post hoc test with  ^*∗*^*p* < 0.05,  ^*∗∗*^*p* < 0.01,  ^*∗∗∗*^*p* < 0.001,  ^*∗∗∗∗*^*p* < 0.0001, ns = nonsignificant.

Cannabis molecules (1–9) (Figure 1(a)) were screened on differentiated Neuro 2a cell line against NMDA-induced excitotoxicity at 10 *µ*M each for 24 hr. MTT assay was used to determine the cell viability [15] and the molecule with the higher cell survival percentage was selected, which was shown by the molecule (9), identified as Cannabispirenone A (Table S1, supporting information).

### 2.5. Cell Culture and Treatments

Neuro 2a cell line was procured from ATCC, seeded, maintained in DMEM supplemented with 10% FBS, 3 mM of sodium pyruvate, 100 units/ml of penicillin, and 100 *µ*g of streptomycin in incubator having atmosphere of 5% CO_2_ at 37°C. After attachment of cells in a monolayer formation, cells were treated with 10 *µ*M of trans retinoic acid (RA) for 5 days to promote differentiation and substantial neurite outgrowth. Now, the cells were pretreated with 5, 10, and 20 *µ*M of molecule (**9**) for 24 hr and then exposed to 2 mM of NMDA for 3 hr.

### 2.6. Cell Viability Measurements

Neuro 2a cells were seeded, differentiated with retinoic acid, in 96-well plates at a density of 1 × 10^5^ cells per mL in DMEM medium. The cells were then treated for 24 hr with various concentrations of compound (9) as indicated and NMDA alone. MK-801 (10 *µ*M) was used as positive control as NMDAR antagonist. MTT colorimetric assay was used to determine cell viability [15]. Here, 2.5 mg/ml of MTT was added to each well for 4 hr and the plate was kept at 37°C. The medium was then removed, and the formazan crystals were dissolved in dimethyl sulfoxide (DMSO). Now, after shaking the plate, the absorbance of formazan solution was measured at 570 nm on Biotek plate reader.

### 2.7. LDH Release Assay

The cytotoxicity level was estimated by the measurement of lactate dehydrogenase (LDH) released from cells using the lactate dehydrogenase (LDH) test kit (Sigma–Aldrich). After treating the cells with molecule (**9**), NMDA, and MK-801, as previously stated, cell-free culture supernatants were collected and incubated at room temperature for 30 min in the dark. The supernatants were measured using a microplate reader at 490 nm, as directed by the LDH assay kit (Sigma–Aldrich).

### 2.8. Determination of Antioxidant Activity

The 2,2-diphenyl-1-picrylhydrazyl (DPPH) strategy is a speedy technique for estimating cell properties that incorporates the utilization of free radicals for evaluating the capability of substances to act as free-radical scavengers (FRS). The DPPH testing procedure is related to the disposal of DPPH, which is a stable-free radical. The free radical DPPH interacts with an odd electron to produce a strong absorbance at 517 nm, resulting in a purple coloration. For example, an antioxidant reacts with DPPH to form DPPHH, which has a lower absorbance than DPPH due to its lower hydrogen content. The antioxidant potential of the molecule (9) was evaluated using DPPH assay. The percentage of inhibition was calculated using ascorbic acid as a positive control and Equation (1):

(1) $$\begin{array}{c} \text{Inhibition (\%)}=\frac{\text{OD of control}-\text{OD of sample}}{\text{OD of control}}\times 100. \end{array}$$

### 2.9. Measurement of Intracellular Calcium and ROS Determination

Calcium measurement was determined by measuring the fluorescence signal using the calcium sensitive indicator fluo-8 AM dye. Differentiated Neuro 2a cells after treatment with the molecule (9), NMDA, and MK-801, as stated previously, were loaded with fluo-8 AM by incubating them at 37°C for 30 min in magnesium-free normal buffer solution, containing fluo-8 AM. The cells were imaged to obtain fluorescent images using EVOS Floid imaging and Biotek Fluorescence microscope. To determine ROS, following drugs treatment, ROS production was quantified using H2DCFDA dye (Sigma–Aldrich, USA). Differentiated Neuro 2a cells were treated with 10 *µ*M of H2DCFDA at 37°C for 30 min. After incubation for 30 min, cells were gently rinsed with PBS, and the fluorescence was quantified with a Biotek fluorescence plate reader and an EVOS FLoid cell imaging station.

### 2.10. Measurement of Mitochondrial Membrane Potential (MMP)

Mitochondrial membrane potential was studied using JC-1 staining. Briefly, differentiated Neuro 2a cells were incubated with different concentrations of molecule (9) for 24 hr and exposed to NMDA (2 mM) for 3 hr. After treatment, cells were incubated with 10 *µ*M of JC-1 dye at 37°C for 35 min and fluorescence intensity was measured using EVOS Floid microscope.

### 2.11. Lipid Peroxidation Measurement

The lipid peroxidation level was determined by measuring the concentration of malondialdehyde (MDA), which is the end product of lipid peroxidation and reacts with TBA to form fluorescence adduct. Differentiated Neuro 2a cells were incubated with different concentrations of molecule (9) for 24 hr and exposed to NMDA (2 mM) for 3 hr. At the end of the treatment, the cells were scraped into TCA (1 ml) and centrifuged at 13000 g at 4°C for 2 min. Now, 2 ml of TBA reagent was added to 500 *µ*l of produced cell homogenates. This mixture underwent a 45-min boil, was cooled, and then centrifuged at 3000 rpm and then the reaction was stopped by cooling in an ice water bath. The absorbance was measured at 535 nM after the supernatant was transferred to 96-well plates.

### 2.12. Measurement of GSH Production

Glutathione (GSH) is the most abundant nonprotein thiol and it is essential for antioxidant defense and maintaining redox homeostasis in neurons. Relative change in intracellular glutathione (GSH) in cells after exposure to molecule **(9)** and NMDA was assayed using Glutathione assay kit (PROMEGA). The reaction is catalyzed by the enzyme glutathione-S-transferase. After incubation at 37°C for 30 min in the dark, the fluorescence intensity was monitored at excitation/emission of 360/460 nM.

### 2.13. Measurement of cAMP Production

The cAMP-Glo assay was used to measure cAMP levels according to the manufacturer's protocol (Promega). Cells were stimulated with molecule (**9**) and NMDA at the appropriate concentration and time. After induction, cells were lysed to release cAMP, and the cAMP detection solution containing protein kinase A was added. After stopping the PKA reaction with Kinase-Glo® Reagent, the residual ATP was detected using a luciferase reaction. Luminescence was observed using a Luminometer. To relate luminescence to cAMP concentrations, a cAMP standard curve was used.

### 2.14. Measurement of ATP Production

Adenosine 5′-triphosphate (ATP) is the central metabolite in cellular energy metabolism, and it is hydrolyzed to ADP and inorganic phosphate to provide free energy in a variety of cellular processes. ATP is also an intracellular signaling molecule and is involved in a variety of important biochemical reactions. When cells die, they stop synthesizing ATP, and the existing ATP pool degrades rapidly. As a result, ATP is widely accepted as a viable cell marker. A greater concentration of ATP indicates a greater number of living cells. Therefore, we have checked the ATP production after treating the differentiated Neuro 2a cells with molecule **(9)** and NMDA, as stated previously. As a result, knowing the ATP concentration within cells is critical for understanding cellular activities. ATP assay was observed using ENLITEN® ATP Assay System kit (Promega), according to the manufacturer instructions.

### 2.15. Cell Lysates Preparation and Western Blotting

After treatment with different concentrations of molecule **(9)** and NMDA, as previously stated, cell lysates were prepared by lysing differentiated Neuro 2a cells for 45 min in ice cold RIPA buffer with 1% protease inhibitor cocktail and 1% phosphatase inhibitor cocktail. After centrifuging the cell suspension at 4°C for 20 min at 14,000 rpm, the supernatant was collected. Bradford reagent was used to estimate protein concentrations. Here, 60 *µ*g of protein was separated on a 10%–12% SDS-PAGE gel and transferred to a polyvinylidene difluoride (PVDF) membrane for western blotting (Millipore, USA). The membrane was blocked for 2 hr in 5% bovine serum albumin (BSA) in TBST at room temperature (RT) after overnight incubation with appropriate primary antibody, NRF-2, anti-CB1, ERK1/2, Akt, PI3K and HRP anti-*β*-actin (Sigma–Aldrich), and antirabbit HRP labelled at 4°C. After incubation with the primary antibody, the membranes were washed three times with TBST for 5 min each time and incubated for 2 hr at RT with horse reddish peroxidase (HRP) conjugated antirabbit (Cell Signaling Technology, USA). The membrane was again washed three times for 15 min each time with TBST. Next, the membrane attached antibodies were observed using Millipore's ECL detection reagent (Billerica, USA). The images were captured using Chemdoc.

### 2.16. Statistical Analysis

All the numerical data were presented as mean ± SD. Statistical analysis of numerical data between glutamate-alone treatment versus glutamate and Cannabispirenone treatment was performed by applying one-way ANOVA analysis.

## 3. Results

### 3.1. Extraction, Isolation, and Characterization of Secondary Metabolites

Flower (0.6 kg) was sequentially extracted with hexane followed by chloroform. The concentrated extracts were combined, and chromatographed by repeated Al_2_O_3_, SiO_2_, and Sephdex LH20 column chromatography. Final purification was achieved by reversed-phase HPLC to give nine compounds **1**−**9**. Based on the HRESIMS data, NMR spectroscopic analysis, and the comparison of the reported NMR data, compounds **1**−**9** were proven to be the known compounds viz 6,12-dihydro-6-hydroxycannabidiverin (**1**), 6,12-dihydro-6-hydroxycannabidiol (**2**) [16], *Δ*^9^-epoxycannabidiol (**3**) [13], 8-hydroxy-isohexahydrocannabinol (**4**) [12], canniprene (**5**) [17], cannabispiran (**6**) [18], 3,9-dihydroxy-4-megastigmene (**7**) [19], 6-epi-Chakyunglupulin B (**8**) [20], and Cannabispirenone A (**9**) [18].

#### 3.1.1. Cannabispirenone A (9)

Colourless crystal; [*α*]_*D*_^25^- AA (c 1.0, MeOH); ^1^H NMR (400 MHz, MeOD) *δ* 6.88 (dd, *J* = 10.1, 1.6 Hz, 1H), 6.20 (d, *J* = 2.0 Hz, 1H), 6.06 (d, *J* = 1.9 Hz, 1H), 5.79 (dd, *J* = 10.1, 0.6 Hz, 1H), 3.60 (s, 3H), 2.90–2.80 (m, 1H), 2.74 (ddd, *J* = 15.9, 8.9, 2.6 Hz, 1H), 2.50 (ddd, *J* = 17.0, 12.9, 5.1 Hz, 1H), 2.41 (dt, *J* = 12.4, 4.2 Hz, 1H), 2.37–2.27 (m, 1H), 2.22 (ddd, *J* = 12.9, 7.7, 2.6 Hz, 1H), 1.93–1.78 (m, 2H); ^13^C NMR (100 MHz, MeOD) *δ* 201.59, 161.00, 160.55, 154.36, 146.13, 125.75, 125.44, 100.63, 99.62, 54.33, 48.25, 35.11, 35.00, 30.71, 30.59; and (+) HRESIMS *m/z* 245.1173 [M+H]^+^ (calculated for C_15_H_17_O_3_^+^ 245.1172).

#### 3.1.2. Cannabispirenone A (9) Molecule Results in Attenuation of NMDA Exposed Cell Death and Results in Reducing the LDH Release in Differentiated N2a Cells against NMDA Exposure

Molecule **(9)** identified as Cannabispirenone A exhibits (84.2 ± 3.4%) cell viability against NMDA-induced excitotoxicity in Neuro 2a cells. Furthermore, Neuro 2a cells, after differentiation and by confirming the expression of differentiation markers MAP2 and *β*-3 tubulin (Figure 1(b)), were pretreated with varying doses of molecule (**9**) before being exposed to NMDA for 2 hr, and the cell death caused by NMDA was greatly reversed. The cell death reversal was 46.2, 62.8%, 84.2%, and 87.3% at 1, 5, 10, and 20 *µ*M concentration of the molecule, respectively (Figure 1(c)). The molecule (**9**) neuroprotective activity was found to be concentration dependent and its effect was more prominent at 10 *µ*M and 20 *µ*M concentration, when compared with the NMDA one (58%), which induces cell death, as measured by MTT assay. Therefore, we observed it clearly that 10 *µ*M and 20 *µ*M concentration of molecule (9) reverses NMDA-induced cell death in differentiated Neuro 2a cells. In addition, the NMDA treatment to the cells results in the disintegration of the membrane integrity to the cell. Here, when we treated cells with NMDA only, the percentage LDH release was 84.2 as compared with the control cells, which significantly reduced when we pretreated the cells with different concentrations of the molecule (9) (Figure 1(d)). It indicates that the molecule is able to reduce the membrane integrity loss to the cell.

#### 3.1.3. 2,2-Diphenyl-1-Picrylhydrazyl Free Radical Scavenging Activities: Antioxidant Potential of the Molecule

The stable radical 2,2-diphenyl-1-picrylhydrazyl (DPPH) has been used to determine the principal antioxidant activity of pure substances or extracts, which reduces the ferric complex into the blue ferrous complex by taking a single electron from an antioxidant. In a cell-free system, molecule (**9**)'s antioxidant capability is demonstrated (Figure 1(e)). The ability to scavenge free radicals and molecule (**9**) 's ferric ion reduction potential is acting as dose dependent. This assay included ascorbic acid as a standard.

#### 3.1.4. The Cannabispirenone A (9) Inhibits Intracellular Calcium Release and ROS Accumulation Induced by NMDA in Differentiated Neuro 2a Cells

In differentiated Neuro 2a cells, NMDA caused a significant release of intracellular calcium (Figures 1(f) and 1(g)). The rising levels of intracellular calcium were dramatically reversed in cells pretreated with MK-801 and different doses of molecule (**9**). The reduction in calcium loads by the molecule (**9**) was significantly comparable with MK-801, a NMDA antagonist. NMDA toxicity results in increase in the regulation of ROS and oxidative stress in the cells. Here, fluorescence microscopy with a DCFH-DA fluorescent probe revealed that NMDA administration resulted in ROS production in differentiated Neuro 2a cells. Fluorescence microscopy revealed that varying doses of molecule (**9**) and MK-801 significantly reduced NMDA-induced ROS production (Figures 1(h) and 1(i)).

#### 3.1.5. Cannabispirenone A (9) Induces Recovery of Membrane Potential of Mitochondria Loss in Excitotoxicity Exposed to NMDA

**Figure 2:**
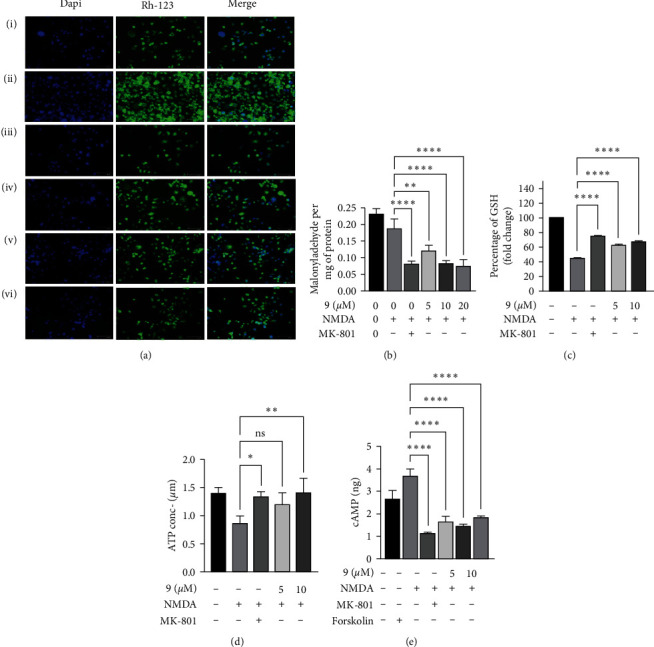
(a) Mitochondrial membrane potential was assessed via live cell imaging. (b) Effect of the molecule (9) on lipid peroxidation in NMDA-induced toxicity. (c) Effect of the molecule (9) on GSH release in NMDA-induced toxicity. (d) Effect of the molecule (9) on intracellular ATP in NMDA-induced toxicity. (e) Effect of the molecule (9) on cyclic AMP release in NMDA-induced toxicity. Data represent an average of three independent experiments. Statistical significance was determined using one-way ANOVA analysis followed by Dunnett's post hoc test with  ^*∗*^*p* < 0.05,  ^*∗∗*^*p* < 0.01,  ^*∗∗∗*^*p* < 0.001,  ^*∗∗∗∗*^*p* < 0.0001, ns = nonsignificant.

Neuro 2a cells after differentiation, NMDA administration caused significant decrease in mitochondrial membrane potential when compared with vehicle (Figure 2(a)). However, membrane potential loss was significantly reduced in cells pretreated with different doses of molecule (**9**) and MK-801, whereas mitochondrial membrane integrity was maintained.

#### 3.1.6. In NMDA-Induced Excitotoxicity, Malondialdehyde Levels Drop by Cannabispirenone A (9)

Malondialdehyde levels (MDA), a byproduct of lipid peroxidation and a critical marker of oxidative damage, were measured to investigate the influence effects of lipid peroxidation on DNA damage caused by free radicals. We discovered that MDA levels in Neuro 2a cells exposed with NMDA were considerably higher (Figure 2(b)) than in untreated cells and that MDA levels were substantially lower in Neuro 2a cells when treated with NMDA antagonist, MK-801 and molecule (**9**).

#### 3.1.7. Cannabispirenone A (9) Increases GSH Levels in NMDA-Exposed Neuro 2a Cells

We investigated whether the antioxidant action of the molecule (**9**) on Neuro 2a cells exposed to NMDA was linked to the regulation of antioxidant enzymes GSH. We have found that GSH activity was reduced in NMDA-treated cells relative to control cells, but this was reversed when the cells were pretreated with molecule (**9**) and MK-801 at the specified dosages (Figure 2(c)). These results suggest that molecule (**9**) may contribute to the ROS scavenging effect.

#### 3.1.8. Cannabispirenone A (9) Potentially Restores the ATP Production in NMDA-Treated Cells

In the presence and absence of NMDA-induced toxicity, the effect of the molecule (**9**) on the ATP content of differentiated Neuro 2a cells was investigated further. As shown in Figure 2(d), the ATP content was reduced in Neuro 2a cells after being treated with NMDA as compared with the control cells. However, the ATP content of the exposed cells increased when the cells were pretreated with different concentrations of molecule (**9**). These findings suggest that the molecule (**9**) can aid mitochondrial function in the face of glutamate shocks.

#### 3.1.9. Cannabispirenone A (9) Potentially Induces the Cyclic AMP (cAMP) Production in NMDA-Treated Cells

As we know cAMP is the key regulator of the neuroprotective component. We investigated the role of molecule (**9**) on the activity of cAMP. Interestingly, we observed that molecule is able to induce cAMP activity in differentiated cells (Figure 2(e)), as compared with the NMDA alone. Forskolin is used as a positive control, which is a potent stimulator of cAMP.

#### 3.1.10. Cannabispirenone A (9) Molecule Resulting in an Increase in Cannabinoid Receptor 1 (CB1) Expression and NRF-2 Expression

**Figure 3:**
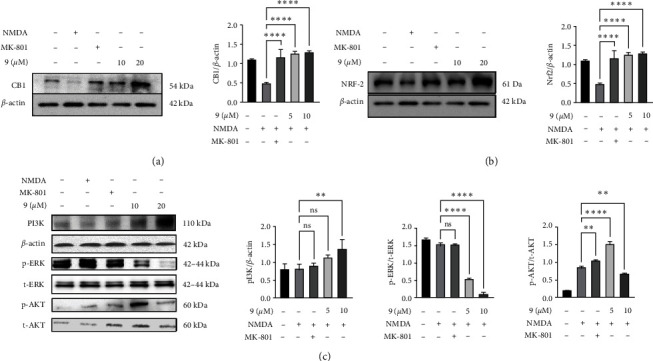
(a) Effect of the molecule (9) on CB1 expression in NMDA-induced toxicity. Representing immunoblots depicting the increase in CB1 expression on treatment of molecule (9). (b) Effect of the molecule (9) on NRF-2 expression in NMDA-induced toxicity. Representing immunoblots depicting the increase in NRF-2 expression on treatment of molecule (9). (c) Effect of the molecule (9) on p-PI3K, p-ERK1/2, and p-Akt expression in differentiated N2a cells against NMDA-induced toxicity. Representing immunoblots depicting their expression pattern on treatment of molecule (9). Data represent an average of three independent experiments. Statistical significance was determined using one-way ANOVA analysis followed by Dunnett's post hoc test with  ^*∗*^*p* < 0.05,  ^*∗∗*^*p* < 0.01,  ^*∗∗∗*^*p* < 0.001,  ^*∗∗∗∗*^*p* < 0.0001, ns = nonsignificant.

Because molecule (**9**) is an analogue of the cannabis plant, we also investigated the expression of the cannabinoids receptor 1, with respect to the treatment of the molecule and interestingly, we have found that molecule results in increase in the expression of cannabinoids receptor 1 (Figure 3(a)), which might be the link with its neuroprotective potential. Since we know, Nrf2 is a critical regulator of endogenous defense system and is actively produced in response to the oxidative stress in the cells. The ability of the molecule **(9)** to activate antioxidant proteins was investigated in differentiated Neuro 2a cells. We found that at 10 and 20 *µ*M concentration, molecule increased Nrf2 expression, suggesting that compound may increase the accumulation of NRF2 in Neuro 2a cells (Figure 3(b)).

#### 3.1.11. Cannabispirenone A (9) Involves the PI3K Signaling, via Rescuing the NMDA-Mediated Insult in Neuro 2a Cells

Immunoblots (Figure 3(c)) depicts the effect of molecule **(9)** on NMDA-induced toxicity mediated changes in the expression levels of p-AKT as well as on p-PI3K proteins. Expression analysis indicated that NMDA treatment led to decrease in p-PI3K expression; however, the pretreatment of molecule **(9)** at 10 and 20 *µ*M concentration rescues the NMDA-mediated toxicity and induces cell survival via increase in p-PI3K expression. Similar expressions of p-AKT were observed, at 10 *µ*M and 20 *µ*M concentration of molecule **(9).** In contrast, NMDA treatment increases p-ERK expression; however, the pretreatment of molecule **(9)** at 10 and 20 *µ*M concentration increases p-ERK expression as compared with NMDA-treated cells. Densitometric analysis revealed a decrease in the expression of p-PI3K and p-AKT on NMDA-mediated toxicity in Neuro 2a cells. Furthermore, treatment with MK-801 and 10 and 20 *µ*M concentration of molecule has reversed the expression of both proteins.

## 4. Discussion and Conclusion

The results of the present study revealed that molecule 9 ameriolated the NMDA-induced death in differentiated Neuro 2a cells. Molecule 9 rescued the cells from NMDA-induced toxicity by abating the LDH levels, abating intracellular calcium levels, attenuating excessive ROS generation, and correcting mitochondrial machinery. Molecule 9 increased the enzymatic defense system in the cells by increasing GSH levels and also increases ATP and cyclic AMP in the differentiated Neuro 2a cells involving activation of NRF-2 protein. We have also found that the molecule 9 increases the CB1 expression providing neuroprotection and activated the PI3K family proteins.

NMDARs (N-methyl-d-aspartate receptors) are a subclass of glutamate receptors that, upon activation, mediate calcium entry and need both glutamate binding and postsynaptic depolarization [21]. Numerous neurological and psychiatric disorders may be exacerbated by NMDAR malfunction, which can result from changes in receptor-channel activity, subunit expression, trafficking, or location. In fact, it is now thought that synaptic dysfunction may be the cause or at least a contributing factor in a wide range of nervous system disorders [22]. NMDA excitotoxicity promotes significant increase in cytosolic calcium concentrations, which activates various pathologic processes such as ROS generation, oxidative stress, ATP depletion, problems in ETC chain, and ultimately results in cell death. It has been reported that calcium influx through NMDAR was associated with significant calcium toxicity in cells [23].

The pharmacological manipulation of ECS is an exciting target for the development of targeted therapies in human disorders [24]. The presence of CB1 receptors in brain cells is essential for direct neuroprotection against a variety of stressors, most notably excitotoxicity. The disruption of the endocannabinoid system in multiple sclerosis has been reported in several papers [25]. All classes of cannabinoids have the ability to guard neurons from a range of insults, including excitotoxicity, calcium influx, free radical production, and neuroinflammation, which are believed to be the cause of delayed neuronal death following traumatic brain injury (TBI) [26].

In present study, we have demonstrated that molecule 9 identified as Cannabispirenone A was able to rescue NMDA-induced cell death in differentiated Neuro 2a cells. To our knowledge, we reported for the first time demonstrating Cannabispirenone A (**9**) has neuroprotective activity against cell death induced by NMDA. Although there are several studies that demonstrate neuroprotective role of cannabinoids [27]. In one of the study, cannabidiol enhanced cell viability and reduced apoptosis in H_2_O_2_-treated nucleus pulposus cells in vitro [28]. In one of the study, authors found that N-linoleyltyrosine protects the neurons against A*β*1–40-induced cell toxicity via autophagy that involves CB2/AMPK/mTOR/ULK1 pathway [29].

We have found significant increase in cytosolic calcium content and ROS generation in NMDA-induced stress in Neuro 2a cells and these increased levels in calcium and ROS generation were attenuated by Cannabispirenone A (**9**) molecule. In addition, in both cell-based and cell-free studies, molecule (**9)** reduced the formation of free radical species, and membrane integrity of mitochondria was restored, all of which had been triggered by NMDA treatment. Calcium release-activated calcium currents are blocked by cannabinoids, which show great effectiveness against SOCE. Specifically, cannabigerolic acid and other carboxylic acid derivatives are particularly effective in this regard. Furthermore, we demonstrate that human T lymphocytes' production of interleukin 2 and nuclear factor of activated T-cell activation decreased as a result of this SOCE inhibition [30]. A type of cannabinoid, CBD operates on the CB1 and CB2 receptors. Because CBD also interacts with other molecular targets, the specific molecular targets behind its many therapeutic effects have not been found. Considering that both CBD and calpain are linked to a number of diseases connected to calcium signaling, including neurological disorders, and that CBD modifies the intracellular calcium level that controls calpain activity [31]. When intracellular calcium levels are out of balance, adverse events happen all at once, including mitochondrial malfunction and the activation of lots of calcium binding proteins. A number of harmful events culminating in mitochondrial dysfunction and the activation of numerous calcium binding proteins are set off by the dysregulation of intracellular calcium levels. However, certain Ca2+ ion levels are first neutralized by mitochondria to prevent a pathological state in a cell; however, continuous Ca2+ inflow results in excessive generation of ROS and oxidative stress; additional evidence indicates that ROS-mediated stress plays a major role in the pathogenesis of many neurodegenerative disorders. Florescence microscopy in the current study revealed that Cannabispirenone A significantly reduced ROS generation. A prior study that demonstrated cannabinoids notable capacity to reduce ROS levels in hypobaric hypoxia supported these findings. It has been discovered that reduction in ROS generation protects the neuron against neurotoxicity [32, 33]. The current findings support our previous report, which demonstrated that the cannabispirenone reduced the generation of reactive oxygen species.

Glutamatergic dysfunction-related neurodegenerative diseases have a common pathogenesis that includes disruption of calcium homeostasis within the cell, nitric oxide synthesis activation, production of free radicals, and programmed cell death leading to progressive neurodegeneration. By potentially opening the mitochondrial permeability transition pore, these mechanisms worsen energy failure and disturb membrane potential by increasing the production of reactive oxygen species. Neural cell death is the result of massive ROS generation and concurrent downregulation of antioxidant mechanisms. Because mitochondria are essential for regulating different cell death pathways and preserving cellular homeostasis, we have examined the impact of cannabispirenone on glutathione enzyme excitotoxicity caused by glutamate. CBD administration to MCT-treated rats increased concentrations in antioxidant compounds TAC and GSH [34].

It also regulates the ATP potential as well as result in elevated levels of cAMP. These findings imply that the brain is protected from damage by activation of mito KATP, which is most likely mediated by reducing mitochondrial Ca2+ overload and preventing MPTP opening during brain ischemia and reperfusion [35]. It is interesting to note that while ATP had no neuroprotective effects in monocultures, it did in astrocyte–neuron cocultures against H_2_O_2_-evoked neuronal cell death [36].

The NMDA currents reduced the cAMP production mediated by forskolin and the treatment of cannabispirenone molecule was able to elevate the cAMP production. The neurotrophic activity of pituitary adenylate cyclase-activating polypeptide (PACAP) and vasoactive intestinal peptide (VIP) is specifically influenced by activation of the adenylyl cyclase (AC)/cyclic AMP (cAMP) signaling pathway [37]. The mechanisms that connect extracellular neuroactive chemicals to cAMP-mediated neuroprotection are likely an important aspect of the nervous system's modulation of sensitivity to cell death [38–40].

A basic-region leucine zipper transcription factor, nuclear factor (erythroid-derived 2)-like 2 (Nrf2), controls the expression of numerous Phase II detoxifying/antioxidant enzymes. Through the Nrf2 pathway, a variety of natural products activate antioxidant enzymes, which in turn has preventive and/or therapeutic effects. In this study, we have found the molecule **(9)** increases the expression of NRF-2 protein, suggesting that the molecule (9) acts as a neuroprotective agent.

Furthermore, we also observed increase in the expression of cannabinoids receptor 1, which regulates neuroprotection and may play a major role in protecting cells against insult. In addition, we report that the Cannabispirenone A from the cannabis plant provide protection to the cells against cell death [21, 41, 42].

However, when considering neurodegenerative diseases, a steady influx of calcium ions causes an increase in reactive oxygen species (ROS) generation and oxidative stress by depleting ATP and disconnecting the electron transport chain and appears to have a substantial role in the etiology of many neurodegenerative diseases [43–45]. NMDAR over activation is believed to the cause of cell death in diseases such as Alzheimer's, Huntington's, and Parkinson's disease [46, 47]. Proper and optimum NMDAR activity is necessary for the neuronal survival and physiological processes, as its excessive stimulation causes pathological activation resulting in cell death [48]. It has been reported that calcium influx through NMDAR channel was associated with significant cell death [4, 9]. *Cannabis sativa* has been among many cultures for its medicinal, pharmacotherapy, and therapeutic potential. It has been used for the treatment of seizures, epilepsy, asthma, skin burn, and in many gastrointestinal disorders [49]. Cannabis constituents have possible therapeutic effects over a broad range of neuronal disorders [50]. It attenuates anxiety and depressive-like behaviors and promotes neurogenesis, anti-inflammatory, anticonvulsive, as well as neuroprotective agent [51, 52]. Researchers are looking into this varied family of chemicals potential as neuroprotective agents due to their capacity to influence neurotransmission and function as anti-inflammatory and antioxidative agents.

In the current study, we assessed the flowers of the cannabis plant that showed ability to protect cells from NMDA-induced insult and discovered that it could prevent cell death. To our knowledge, we here reporting the first-time neuroprotective properties of the molecule isolated from the flowers of the cannabis plant. In one of the study, nine cannabinoid compounds have been shown to induce neuroprotective in MC65 cells [53]. Oleamide, having endocannabinoid profile, has shown protective properties in rat brain against quinolinic acid-induced excitotoxicity. In the study, authors observed that oleamide prevented the deficiency of mitochondrial limit in synaptosomes in a system intervened by the CB1 receptor [54]. Furthermore, cannabinoids also reduces the inflammatory activity of microglia and improves neuronal survival [55]. Cannabinoids also improve cell survival against *β*-amyloid toxicity in rat hippocampal neurons via the reduction of oxidative stress, inflammation, and Nrf2 activation [56].

After inducing NMDA to the cells, we observed considerable increase in intracellular calcium currents and these calcium currents were reduced by the different concentrations of cannabinoid molecule, supported by other researchers [57]. The major influx of calcium ions leads to excessive generation of reactive oxygen species, oxidative stress, and ultimately cell death suggesting that ROS may be the major player in inducing cell death in many neurodegenerative disorders. In the current study, cannabinoid molecule also reduces reactive oxygen species as observed by fluorescence microscopy. We have also found increase in the cannabinoid receptor 1 expression after treating with different dosages of cannabinoid compound, supported by other study [54]. We also discovered a significant decrease in NRF-2 expression levels following exposure to NMDA, which was significantly reversed by pretreating the cells with various cannabis compound dosages. We have also found that the molecule is able to increase expression of p-PI3K and p-Akt and decrease p-Erk1/2 protein levels as compared with the NMDA exposure. ERK1/2 function as important protein kinases as they regulate diverse cellular activities including neuronal survival and differentiation, we here observed decrease in p-ERK1/2 expression contribute to the survival signal to the cells. In conclusion, we found that the molecule (9) can protect neurons from excitotoxic insult through PI3K/Akt signaling pathway.

We believe that the current findings are promising, but they must be extensively verified in primary neuronal cultures. Our future research will focus on determining suitable molecular mechanism to Cannabispirenone A (**9**) molecule activity as well as pharmacokinetics and administration choices in animal models.

## Data Availability

All data generated in this work are included in the figures of the manuscript. Further enquiries can be directed to the corresponding author.

## Supplementary Materials

Supplementary Materials Figure S1: ^1^H NMR (MeOD, 400 MHz) of Cannabispirenone A (**9**). Figure S2: 13C NMR (MeOD, 400 MHz) of Cannabispirenone A (9). Figure S3: DEPT-135 NMR (MeOD, 400 MHz) of Cannabispirenone A (9). Figure S4: HPLC chromatogram for the separation of compounds 1–6. Figure S5: standard HPLC chromatogram for the separation of compounds 1–6. Table S1: percentage cell survival of the compounds against NMDA-induced cell death in differentiated Neuro 2a cells.
