# Supplementary material for: Neuroprotective Effects of Cannabispirenone A against NMDA-Induced Excitotoxicity in Differentiated N2a Cells
Source: Oxid Med Cell Longev. 2024 Feb 13;2024:3530499. doi: 10.1155/2024/3530499 (PMC11161259; doi:10.1155/2024/3530499)
Supplement: Supplementary Materials — Figure S1: 1H NMR (MeOD, 400 MHz) of Cannabispirenone A (9). Figure S2: 13C NMR (MeOD, 400 MHz) of Cannabispirenone A (9). Figure S3: DEPT-135 NMR (MeOD, 400 MHz) of Cannabispirenone A (9). Figure S4: HPLC chromatogram for the separation of compounds 1–6. Figure S5: standard HPLC chromatogram for the separation of compounds 1–6. Table S1: percentage cell survival of the compounds against NMDA-induced cell death in differentiated Neuro 2a cells. [file 3530499.f1.pdf]

Supplementary Information

Neuroprotective Effects of Cannabispirenone A Against NMDA-Induced Excitotoxicity in Differentiated N2a Cells

Sonia Thapa[1,4], Yedukondalu Nalli[2], Ajeet Singh [1,4], Shashank Kr. Singh[1,*], Asif Ali[2,3,*]

[1]*Cancer Pharmacology Division, CSIR-Indian Institute of Integrative Medicine, Canal Road, Jammu, 180001, India*

[2]*Natural Products Chemistry Division, CSIR–Indian Institute of Integrative Medicine, Canal Road, Jammu Tawi, 180 001, India*

[3]*Medicinal and Process Chemistry Division, CSIR-Central Drug Research Institute, Lucknow 226031, India*

[4]*Academy of Scientific and Innovative Research (AcSIR), Ghaziabad, India*

**Corresponding Author*

SKS, Contact number: +919469709444, Email: sksingh@iiim.ac.in; A.Ali Contact number: +919419273507, Email: asifali@cdri.res.in.

Table of contents

Figure S1: ^{1}H NMR (MeOD, 400 MHz) of Cannabispirenone A (**9**)

Figure S2: ^{13}C NMR (MeOD, 400 MHz) of Cannabispirenone A (**9**)

Figure S3: DEPT-135 NMR (MeOD, 400 MHz) of Cannabispirenone A (**9**)

Figure S4: HPLC chromatogram for the separation of compounds **1-6**

Figure S5: Standard HPLC chromatogram for cannabispirenone A (9)

Table S1: Percentage cell survival of the compounds against NMDA-induced cell death in differentiated Neuro2a cells.

Figure S1: ^{1}H NMR (MeOD, 400 MHz) of Cannabispirenone A (**9**)

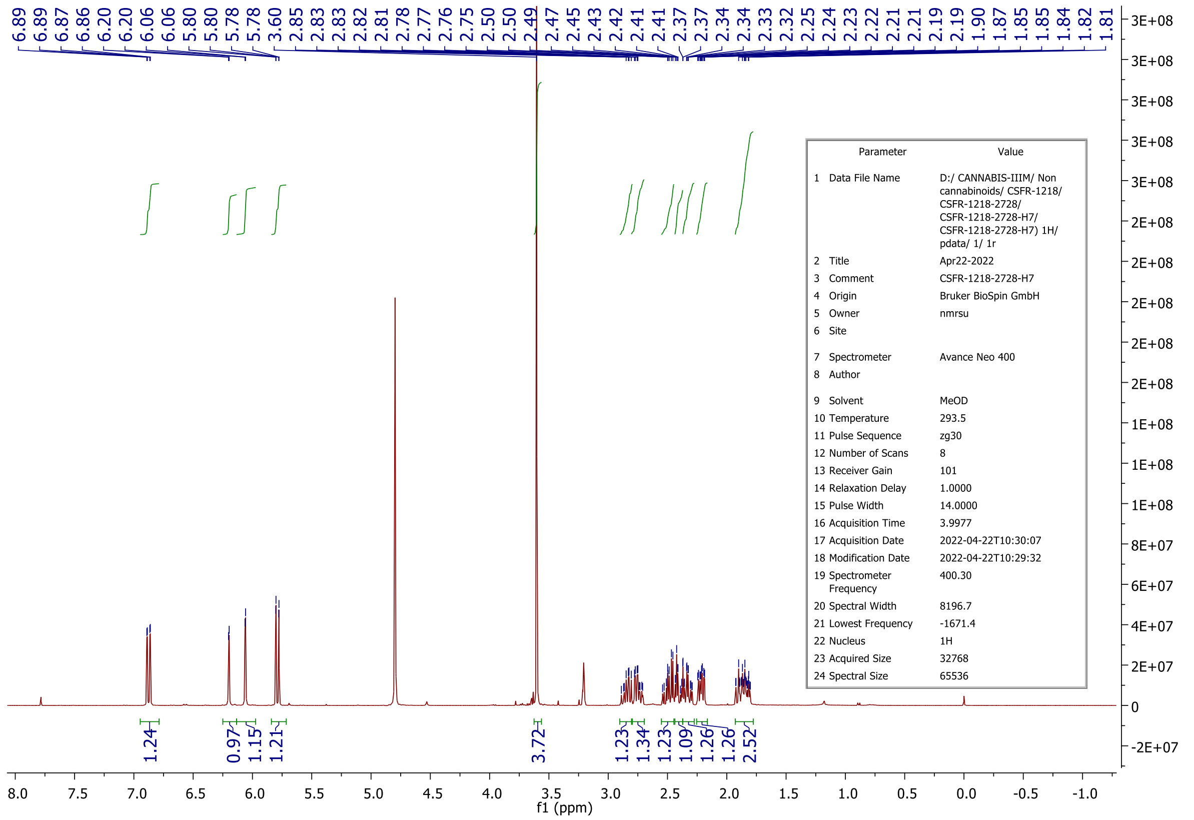

Figure S2: ^{13}C NMR (MeOD, 400 MHz) of Cannabispirenone A (**9**)

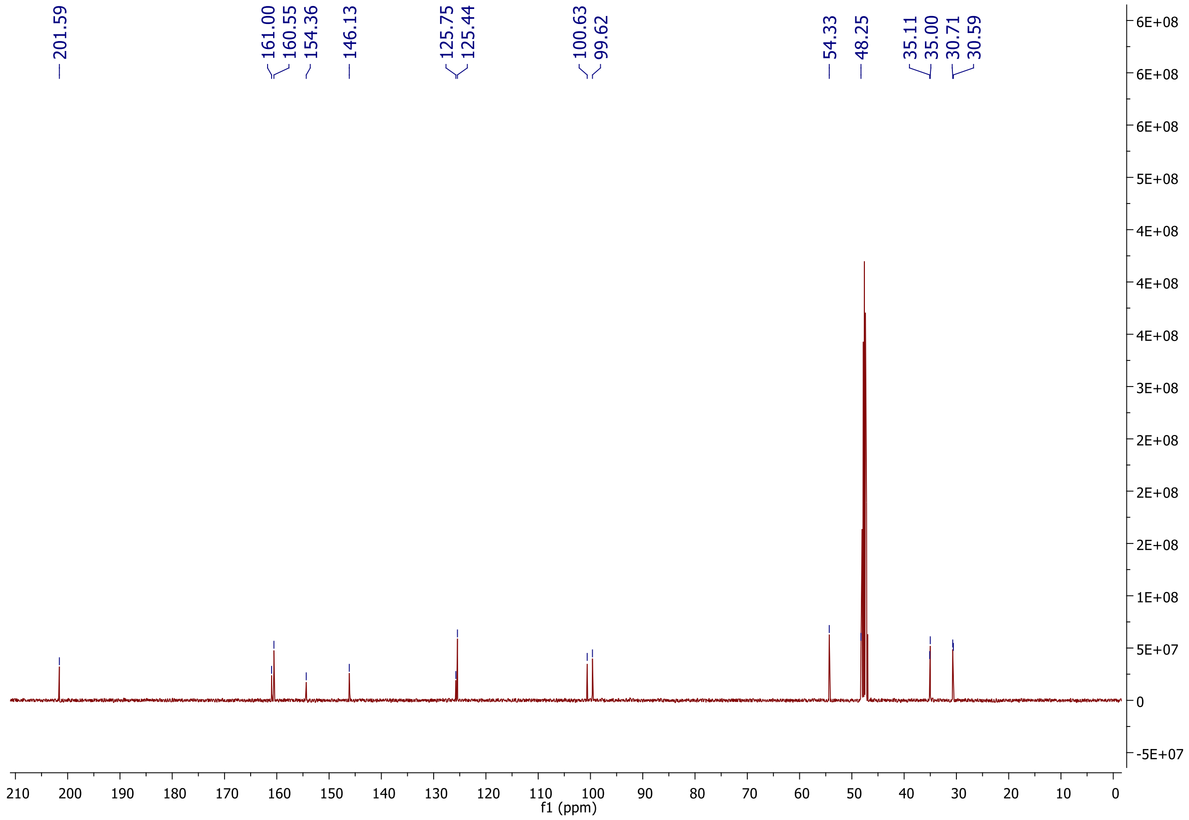

Figure S3: DEPT-135 NMR (MeOD, 400 MHz) of Cannabispirenone A (**9**)

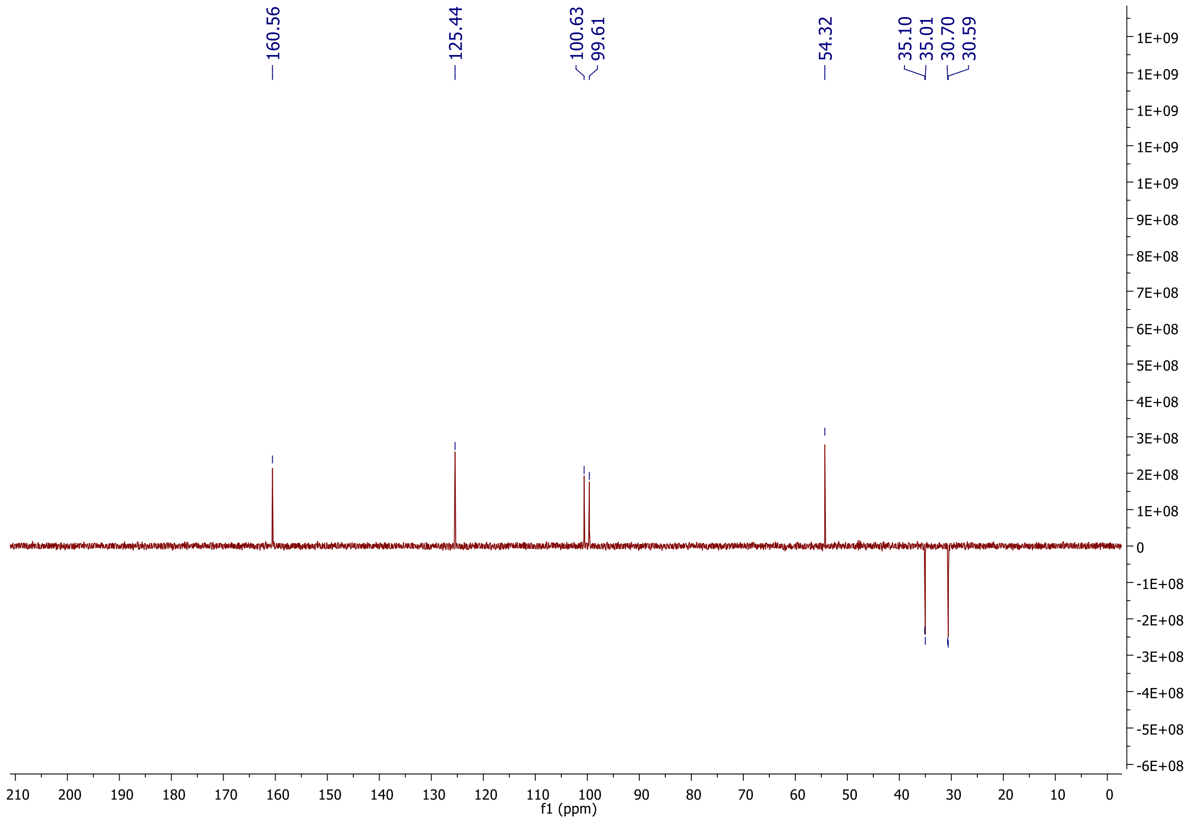

Figure S4: HPLC chromatogram for the separation of compounds **1-6**

flowers
↓ Hexane
Hexane Ext (61.7g)
↓ alumina
Fraction [0.92g]
294-309

Natural Product Chemistry

USER:YEDU
Data file:D:\Agilent Technologies\Result\YEDU\CS-LDK.rslt\1-Rep1
Method Name:D:\Agilent Technologies\Result\YEDU\CS-LDK.rslt\CS-LDK.met
5/12/2018 8:25:24 AM (GMT +05:30)
Sample ANSHU (CS-FRACTION-1)
Injection Vol.: 5 uL
METHOD;

TIME	0	20
C8(ACN in WATER)	80	80

FLOW RATE; 2.0 mL/min, COLOUMN;Agilent Eclipse XDB-C18, 5 UM, 9.4X250 mm

0.139 gms

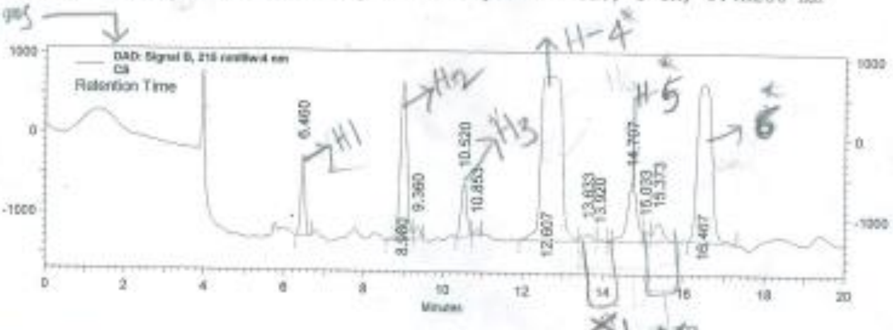

DAD: Signal B,
215 nm/Bw:4 nm
Results

Peak Number	Retention Time	Area	Area Percent
1	6.460	14743524	2.464
2	8.980	47844847	7.997
3	9.360	2725599	0.456
4	10.520	13844832	2.314
5	10.853	1353153	0.226
6	12.607	152988706	25.572
7	13.633	2679345	0.448
8	13.920	633719	0,106
9	14.707	25864646	4.323
10	15.033	3095638	0.517
11	15.373	7837866	1.310
12	16.467	102584707	17.147
13	25.133	162546409	27.170
14	26.467	1553546	0.260
15	26.647	2398199	0.401
16	29.320	15107024	2.525
17	29.793	16543195	2.765
18	30.047	5859208	0.979
19	30.373	6182889	1.033
20	30.567	3220053	0.538
21	30.867	4656148	0.778
22	31.193	3994711	0.668
Totals		598257964	100.000

H1 → 3.1 mg
H2 → 7.7 mg
H3 → 2.3 mg
H4 → 29.6 mg
H6 → 12.0 mg
H8 → 7.9 mg

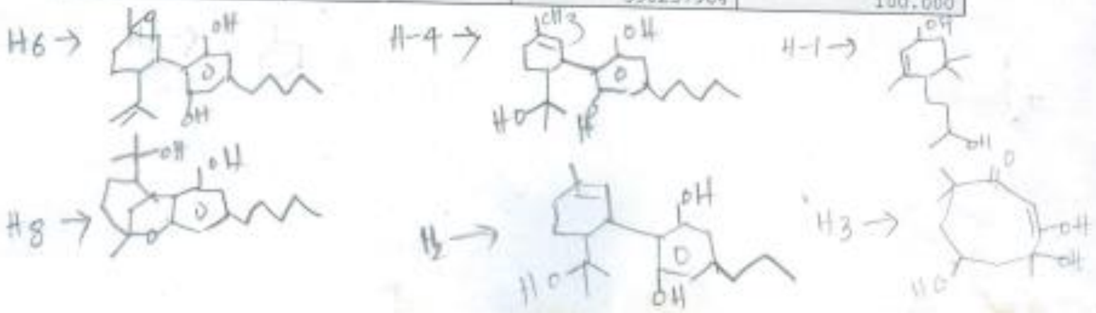

Figure S5: Standard HPLC chromatogram for cannabispirenone A (9)

Empower 3 SOFTWARE

Default Individual Report

SAMPLE INFORMATION

Sample Name:	CSF-1218-2728-7	Acquired By:	System
Sample Type:	Unknown	Sample Set Name:	CS1218
Vial:	1:B,3	Acq. Method Set:	CS1218
Injection #:	1	Processing Method:	MKV_Immuno_PM
Injection Volume:	5.00 ul	Channel Name:	2998 Ch3 254nm@4.8nm
Run Time:	60.0 Minutes	Proc. Chnl. Descr.:	2998 Ch3 254nm@4.8nm
Date Acquired:	15-01-2022 03:05:41 IST		
Date Processed:	15-01-2022 14:55:32 IST		

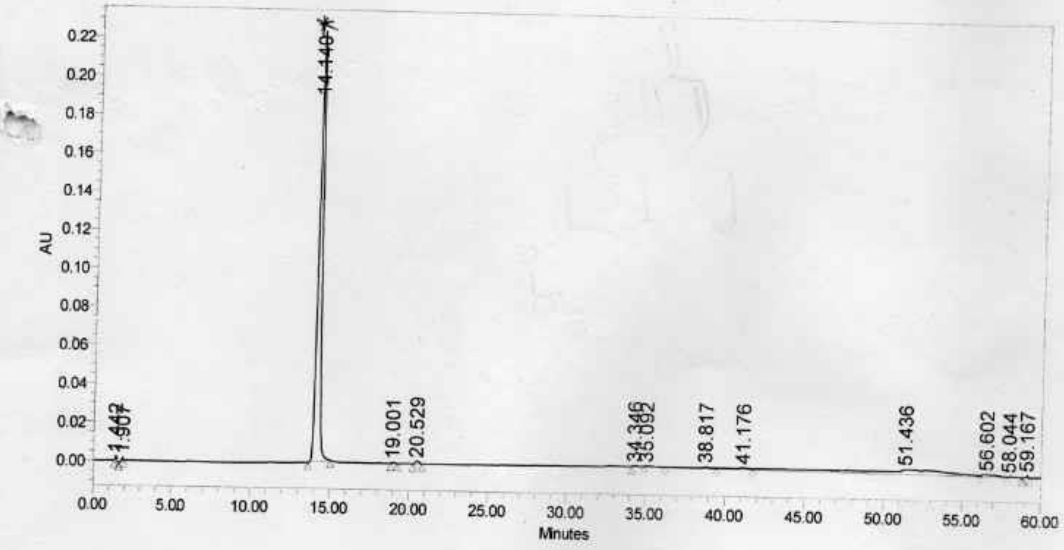

	RT	Area	% Area	Height
1	1.442	18515	0.37	2796
2	1.907	14653	0.29	1918
3	14.140	3520093	70.24	223480
4	19.001	4479	0.09	388
5	20.529	20856	0.42	1457
6	34.346	10852	0.22	532
7	35.092	22926	0.46	519
8	38.817	66658	1.33	960

Reported by User: System
Report Method: Default Individual Report
Report Method ID 1020
Page: 1 of 2

Project Name: 2019\MKV
Date Printed:
15-01-2022
14:55:37 Asia/Calcutta

Table S1: Percentage cell survival of the compounds against NMDA-induced cell death in differentiated Neuro2a cells at 10µM each.

Compound	% Cell viability
**1**	39.4±1.8
**2**	52.1 ± 2.6
**3**	62.2±1.9
**4**	17.6 ± 1.5
**5**	34.8±2.4
**6**	32.9±2.2
**7**	54.2±1.6
**8**	48.7±1.4
**9**	84.2±3.4
